# Sport-Related Concussion Alters Indices of Dynamic Cerebral Autoregulation

**DOI:** 10.3389/fneur.2018.00196

**Published:** 2018-03-27

**Authors:** Alexander D. Wright, Jonathan D. Smirl, Kelsey Bryk, Sarah Fraser, Michael Jakovac, Paul van Donkelaar

**Affiliations:** ^1^MD/PhD Program, University of British Columbia, Vancouver, BC, Canada; ^2^Southern Medical Program, Reichwald Health Sciences Centre, University of British Columbia Okanagan, Kelowna, BC, Canada; ^3^Experimental Medicine Program, Faculty of Medicine, University of British Columbia, Vancouver, BC, Canada; ^4^School of Health and Exercise Sciences, University of British Columbia Okanagan, Kelowna, BC, Canada

**Keywords:** cerebral blood flow, autoregulation, blood pressure, autonomic dysfunction, transfer function analysis, repetitive head impact exposure

## Abstract

Sport-related concussion is known to affect a variety of brain functions. However, the impact of this brain injury on cerebral autoregulation (CA) is poorly understood. Thus, the goal of the current study was to determine the acute and cumulative effects of sport-related concussion on indices of dynamic CA. Toward this end, 179 elite, junior-level (age 19.6 ± 1.5 years) contact sport (ice hockey, American football) athletes were recruited for preseason testing, 42 with zero prior concussions and 31 with three or more previous concussions. Eighteen athletes sustained a concussion during that competitive season and completed follow-up testing at 72 h, 2 weeks, and 1 month post injury. Beat-by-beat arterial blood pressure (BP) and middle cerebral artery blood velocity (MCAv) were recorded using finger photoplethysmography and transcranial Doppler ultrasound, respectively. Five minutes of repetitive squat–stand maneuvers induced BP oscillations at 0.05 and 0.10 Hz (20- and 10-s cycles, respectively). The BP–MCAv relationship was quantified using transfer function analysis to estimate *Coherence* (correlation), *Gain* (amplitude ratio), and *Phase* (timing offset). At a group level, repeated-measures ANOVA indicated that 0.10 Hz *Phase* was significantly reduced following an acute concussion, compared to preseason, by 23% (−0.136 ± 0.033 rads) at 72 h and by 18% (−0.105 ± 0.029 rads) at 2 weeks post injury, indicating impaired autoregulatory functioning; recovery to preseason values occurred by 1 month. Athletes were cleared to return to competition after a median of 14 days (range 7–35), implying that physiologic dysfunction persisted beyond clinical recovery in many cases. When comparing dynamic pressure buffering between athletes with zero prior concussions and those with three or more, no differences were observed. Sustaining an acute sport-related concussion induces transient impairments in the capabilities of the cerebrovascular pressure-buffering system that may persist beyond 2 weeks and may be due to a period of autonomic dysregulation. Athletes with a history of three or more concussions did not exhibit impairments relative to those with zero prior concussions, suggesting recovery of function over time. Findings from this study support the potential need to consider physiological recovery in deciding when patients should return to play following a concussion.

## Introduction

Sport-related concussion is a global public health concern, with recent reports estimating incidences of 1.1–1.9 million injuries each year in US youth alone (1). Whereas the majority of patients recover clinically within 2 weeks (2), concussions are characterized by a period of increased cerebral vulnerability post injury, whereby the brain is more sensitive to additional trauma (2–4). Additional research has shown that the effects of concussion may be cumulative, with reports demonstrating that collegiate athletes with a history of three or more concussions exhibit a threefold increased risk of sustaining a subsequent concussion (5).

The increased cerebral vulnerability observed post concussion may reflect a fundamental difference between *physiological* recovery and *clinical* recovery—heavily based on medical symptom resolution, which guides return-to-learn/return-to-play decisions. Studies have reported alterations in physiological parameters that persist far beyond the typical 7–10 days to clinical recovery. Among other physiological markers, cerebral blood flow (CBF) (6), myelin content (7), and cerebral metabolites (4) have demonstrated recovery profiles on the order of 30+ days.

Alterations in CBF are thought to play an important role in the pathophysiology underlying concussions, though the mechanisms underlying CBF changes are poorly understood (8). In both pediatric and adult patients, local and global reductions in CBF have been reported following sport-related concussion that persists in some cases beyond 30 days (6, 9). Meier and coworkers (6) reported regional reductions in CBF at 1 month in slower-to-recover athletes that were related to initial symptom severity. Contrastingly, a recent study from Barlow and colleagues reported the opposite trend; participants who were asymptomatic at 1 month post injury exhibited reduced CBF compared to non-injured controls, whereas symptomatic participants displayed an increased CBF compared to controls (10). Despite the demonstrated CBF alterations following concussion in the literature, surprisingly little work has focused on the effects of sport-related concussion on CBF control mechanisms.

Concussion-induced CBF disturbances may arise from an alteration in the ability of the cerebral blood vessels to buffer changes in blood pressure (BP)—commonly referred to as cerebral autoregulation (CA) (11). Normally, the cerebrovasculature behaves as a high-pass filter, whereby higher-frequency BP oscillations (>0.20 Hz) are linearly transferred to the cerebral blood vessels, while lower-frequency oscillations are dampened more efficiently (11). Myogenic, neurogenic, and metabolic mechanisms are known to be involved in CA, which functions to protect against surges in BP during periods of relative hypertension and against ischemia during hypotensive episodes (12). Disrupted CA has been hypothesized as a potential mechanism underlying persistent post-concussive symptoms (13). Impairments in CA have been consistently documented following moderate and severe TBI (14, 15), with compromised CA being a significant predictor of poor outcome acutely following severe TBI (16). In more severe TBI, CA is typically assessed using intracranial pressure measures in response to changes in BP to derive indices such as the pressure reactivity index [e.g., (16)]. By contrast, transcranial Doppler ultrasound is an alternate method to assess CBF studies in participants with mild TBI or sport-related concussion. Using this approach, impaired CA has been observed in a small number of hospitalized mild TBI patients, the majority of whom also had abnormal intracranial findings on CT (17, 18). In the context of sport-related concussion, it has previously been demonstrated that a relationship exists between exercise-induced increases in CBF and exacerbations in headache scores. However, very little is known to what extent the dynamic, frequency-dependent relationship between BP and CBF is affected by sport-related concussions (19).

Accordingly, our objectives in the current study were to obtain pre- and post-injury data to provide the first evaluation of the acute and cumulative effects of sport-related concussion on indices of dynamic CA. Toward this end, we sought to examine (i) the effects of acutely diagnosed concussion on the frequency-dependent cerebral pressure flow relationship and (ii) the effect of multiple (3+) previous concussions on CA in otherwise healthy and fully recovered athletes prior to their athletic season.

## Materials and Methods

### Study Design

One hundred and seventy-nine male (mean age 19.6 ± 1.5 years) elite junior hockey (*n* = 90) and American football (*n* = 89) players were recruited for the study. All participants underwent preseason baseline testing. Upon enrollment, 42 players had experienced zero self-reported concussions (Hx^−^) while 31 had sustained three or more self-reported concussions (Hx^3+^). Many previous studies examining the effect of concussion history have compared participants with 1 + vs. no concussions. Others have made the comparison between multiple vs. no concussions [e.g., Ref. (20, 21)], and we decided to use this approach in the present study. Athletes who were subsequently diagnosed with a concussion (*n* = 18) by an independent physician during their competitive season, based on criteria outlined in the Fourth Consensus Statement on Concussion in Sport (2), underwent additional laboratory testing at 72 h, 2 weeks, and 1 month after the injury. Prior to all physiological testing, participants completed the Sport Concussion Assessment Tool, version 3 (SCAT3) (2). The SCAT3 is a concussion screening tool that is composed of characterizing symptom burden, a set of questions termed the Standardized Assessment of Concussion (SAC) that probes orientation, immediate and delayed recall, and concentration, as well as performance on the modified Balance Error Scoring System (BESS). *A priori* exclusion criteria included a significant history of cardiorespiratory, cerebrovascular, neurological, or severe neurodevelopmental disorder; no participants were excluded from this study based on these grounds. All subjects underwent familiarization of the testing procedures and abstained from exercise, caffeine, and alcoholic beverages for at least 12 h prior to all testing sessions (22, 23) All participants provided written informed consent prior to participation in the study, which was approved by the University of British Columbia Clinical Research Ethics Board.

### Instrumentation

Participants were equipped with a three-lead electrocardiogram for measurement of R–R interval and heart rate. CBF was indexed using transcranial Doppler ultrasound (ST3, Spencer Technologies, Seattle, WA, USA) to record blood velocity in the middle (MCAv) cerebral artery on either the right or the left side. After the vessel was identified and signals optimized according to depth, waveform, and velocity, the ultrasound probes were locked in place with a fitted head frame (Spencer Technologies, Seattle, WA, USA). Beat-to-beat BP was recorded with finger photoplethysmography (Finometer PRO, Finapres Medical Systems, Amsterdam, The Netherlands), and partial pressure of expired carbon dioxide (P_ETCO2_) was monitored using an online gas analyzer (ML206, AD Instruments, Colorado Springs, CO, USA). All data were sampled at 1,000 Hz (PowerLab 8/30 ML880, AD Instruments) and stored for offline analysis using commercially available software (LabChart version 7.1, AD Instruments).

### Experimental Protocols

All visits to the laboratory occurred at the same time of the day (22) and involved a hemodynamic challenge protocol (repetitive squat–stand maneuvers) (23). Data were recorded while standing quietly for 5 min to obtain baseline values. During squat–stand maneuvers, subjects started from standing, squatted to and held a ~90° knee angle, and returned to standing at a pace dictated by a metronome. Squat–stands were performed for 5 min at two different frequencies: 0.10 Hz (5-s squatting, followed by 5-s standing) and 0.05 Hz (10-s squatting, followed by 10-s standing). These frequencies were selected to be within the range at which CA is believed to exert the greatest influence on cerebral pressure flow dynamics (11) and are thought to reflect myogenic (0.05 Hz) and autonomic (0.10 Hz) contributions toward CA (24, 25).

### Data Processing

Beat-to-beat mean values of BP (MAP), MCAv, and P_ETCO2_ were determined from each R–R interval. All data were processed and analyzed with custom-designed software in LabView 14 (National Instruments, Austin, TX, USA), as outlined previously (23).

### Power Spectrum and Transfer Function Analysis

Beat-to-beat BP and MCAv data were spline-interpolated and resampled at 4 Hz. In accordance with the recently published best-practice guidelines for transfer function analysis (26), each 5-min recording was divided into five successive windows with 50% overlap. Data within each window were linearly detrended and passed through a Hanning window prior to fast Fourier transform. Transfer function analysis involved determining the cross-spectrum between MAP and MCAv, divided by the MAP autospectrum, in order to derive transfer function *Coherence, Phase*, and *Gain*. Similar to a correlation coefficient, *Coherence* describes the proportion of variance in the output signal (MCAv) explained by the input signal (MAP); a high coherence enables reliable interpretation and improves within-subject stability of *Phase* and *Gain* (23). *Phase* represents the timing offset between input and output oscillations, whereas *Gain* provides a ratio of output amplitude to input amplitude. In the setting of intact CA, a higher *Phase* offset indicates a more rapid alteration in cerebrovascular resistance to a change in BP. Conversely, low gain indicates low magnitudes of BP oscillation being transferred to the cerebrovasculature (23). Metrics were sampled at the point estimate of the driven frequency (0.05 or 0.10 Hz); these point estimates were selected as they fall within the very low frequency (0.02–0.07 Hz) and low frequency (0.07–0.20 Hz) ranges at which CA behavior is believed to be operant (11, 27). Phase wraparound was not present for any of the point estimates at 0.05 or 0.10 Hz.

### Statistical Analyses

All statistical analyses were performed using SPSS Statistics for Macintosh (Version 22.0, IBM Corp., Armonk, NY, USA). Shapiro–Wilks tests were used to assess for normality, while Mauchly’s test was used to evaluate sphericity. In cases where the assumption of sphericity was violated, Greenhouse–Geiser epsilon was used to adjust degrees of freedom for the primary *F*-test. Significance was determined *a priori* to achieve an experiment-wide α = 0.05.

#### Effect of Acute Concussion

A 2 (frequency: 0.05 and 0.10 Hz) by 4 (time: preseason baseline, 72-h, 2-weeks, and 1-month) two-way repeated-measures ANOVA was used to evaluate the effect of acute sport-related concussion on transfer function metrics at each driven frequency. We chose to use a repeated-measures baseline-retest design for this analysis instead of non-concussed controls because it allows a more direct comparison within individual participants and controls for inter-individual variability. Control data in healthy individuals are available in the literature (23), and the baseline measures in our participants were within the normal range for *Coherence, Phase*, and *Gain*. When omnibus tests indicated significant main effects, pre-planned *t*-tests with Bonferroni correction were used to evaluate specific pairwise contrasts (i.e., each post-injury time point relative to baseline). For dependent variables exhibiting a significant effect of time, secondary exploratory analyses were conducted to estimate the relationship between change in CA metrics and change in SCAT3 performance. Specifically, Pearson or Spearman correlation coefficients were calculated between acute change scores (from preseason) in the dependent variable and changes in symptom number, symptom severity, and performance on the SAC and BESS, as recorded within the SCAT3.

#### Cumulative Effect of Multiple Concussions

The cumulative effect of concussions on transfer function metrics was evaluated using a 2 (frequency) × 2 (Hx^−^ vs. Hx^3+^) two-way mixed ANOVA.

## Results

Demographic characteristics, SCAT3 performance, return-to-play durations, and resting physiological data across testing sessions and groups are outlined in Table 1. At preseason, Hx^3+^ participants reported a greater number (*p* = 0.003) and severity (*p* = 0.002) of symptoms than Hx^−^ participants. The resting physiological variables did not differ between these two groups (*p* > 0.05). Athletes who sustained a concussion during the season were cleared by their team physician to return-to-full contact, game participation, a median of 14 days post injury. On average, the concussed athletes had sustained 1.9 concussions previously (range 0–5). A one-way analysis of variance revealed a significant effect of time for the number (*p* < 0.001) and severity (*p* < 0.001) of symptoms and the SAC score (*p* < 0.027) for the athletes who were concussed during the season. Bonferroni-corrected *t*-tests demonstrated that these effects were each due to changes with respect to preseason baseline at the 72-h time point (*p* < 0.05).

**Table 1:** Demographics, SCAT3 performance, and resting physiological parameters at preseason for athletes with no previous concussions or 3+ previous concussions (*left side*) and during each test session for acutely concussed athletes (*right side*).

Metric	Concussion history		*p*	≥Concussed (*n* = 18)				*p*
	0 (*n* = 42)	3+ (*n* = 31)		Preseason	72 h	2 Weeks	1 Month	
Age (years)	19.0 (1.4)	19.6 (1.9)	0.069	18.6 (1.5)				
BMI (kg/m^2^)	27.6 (5.1)	27.5 (4.3)	0.665	25.5 (3.1)				
# Previous Conc	0 (0)	4.5 (2.4)	**<0.001**	1.9 (1.7), range 0–5				
RTP (days)	N/A (preseason only)			median = 14, range 7–35 days				
No. Symptoms	2.3 (2.9)	4.7 (4.3)	***0.003***	2.0 (3.6)	11.2 (5.5)*	2.8 (3.0)	1.1 (2.1)	**<*0.001***
Symptom Severity	3.5 (5.2)	9.3 (12.0)	***0.002***	3.6 (3.6)	25.1 (19.0)*	3.6 (3.5)	1.3 (2.3)	**<*0.001***
SAC Score	27.1 (1.7)	26.7 (1.8)	0.193	26.8 (1.7)	26.5 (2.0)*	26.9 (2.9)	28.1 (1.3)	***0.027***
BESS Score	3.0 (2.6)	3.5 (3.3)	0.793	2.6 (2.6)	4.2 (3.1)	3.0 (2.3)	2.0 (2.0)	0.183
MAP (mmHg)	95.3 (13.8)	94.1 (12.4)	0.838	92.3 (20.1)	90.4 (18.2)	92.0 (16.5)	91.2 (15.9)	0.389
MCAv (cm/s)	55.3 (9.2)	55.6 (9.3)	0.183	53.8 (7.0)	53.5 (8.7)	53.3 (9.5)	53.2 (7.8)	0.435
HR (bpm)	74.7 (9.6)	73.6 (8.7)	0.211	79.4 (8.3)	79.7 (12.2)	82.1 (15.9)	79.5 (10.2)	0.112
P_ETCO2_ (mmHg)	37.9 (2.7)	38.1 (2.5)	0.853	37.7 (3.2)	37.6 (3.7)	37.9 (4.1)	37.6 (4.5)	0.563

*Components of the SCAT3 include symptom number and severity, Standardized Assessment of Concussion (SAC), and Balance Error Scoring System (BESS)*.

*Data are presented as mean (SD) unless otherwise noted*.

*Significant difference from preseason (Bonferroni-corrected t-test).

### Effect of Acute Concussion

**Figure 1:**
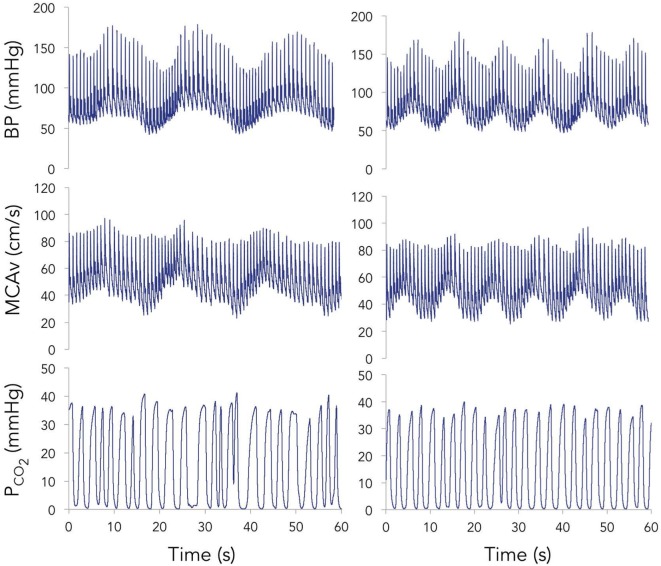
Representative time-series for blood pressure (BP) (top), middle cerebral artery blood velocity (MCAv) (middle), and expired carbon dioxide $\left({\text{P}}_{{\text{CO}}_{2}}\right)$ (bottom) during 60 s of squat–stand maneuvers performed at 0.05 (left) and 0.10 Hz (right).

**Figure 2:**
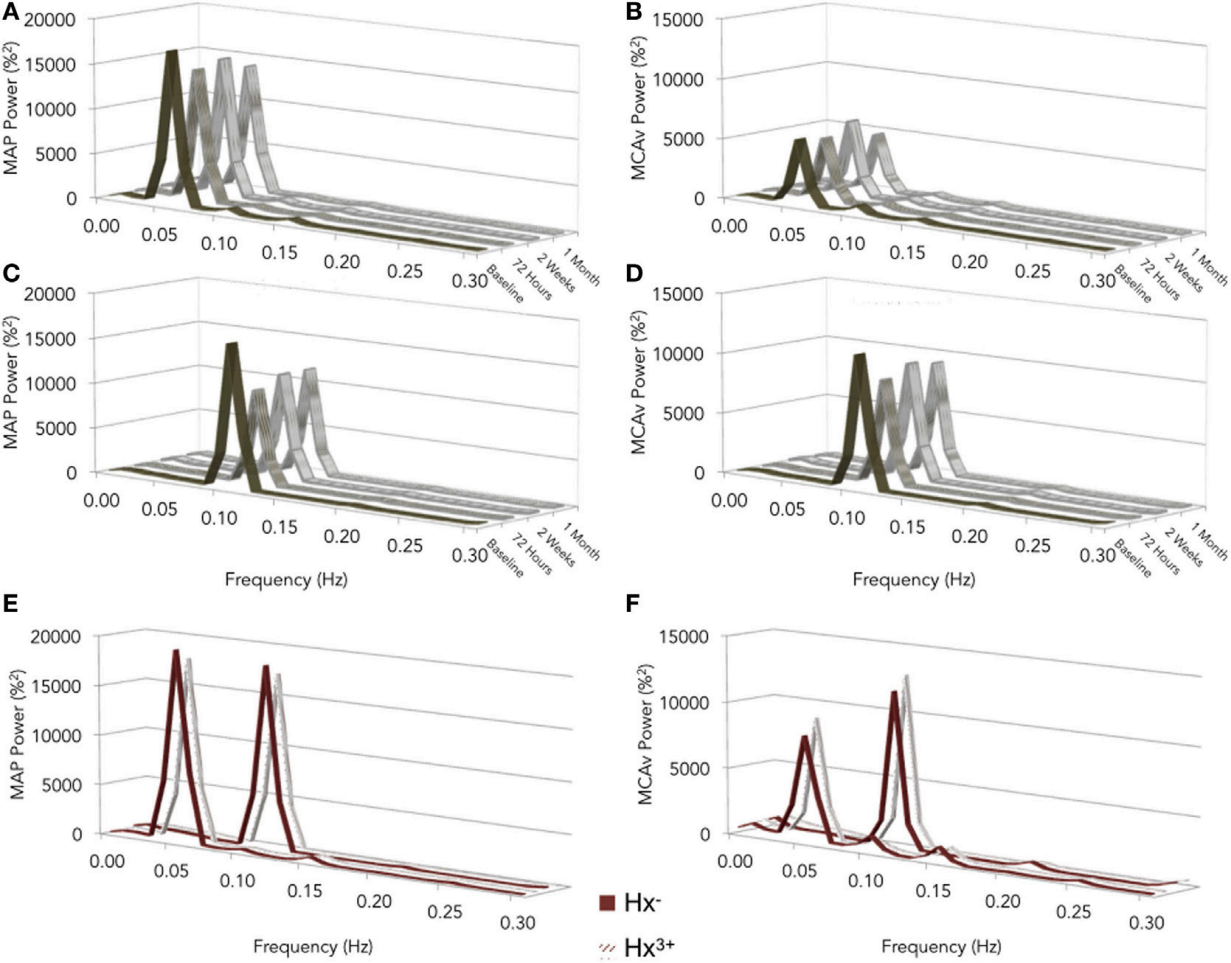
Normalized values of power spectrum densities for mean arterial pressure (MAP) **(A,C)** and middle cerebral artery blood velocity (MCAv) **(B,D)** for preseason and post-concussion squat–stands at 0.05 **(A,B)** and 0.10 Hz **(C,D)**; preseason squat–stands in subjects with zero vs. three or more previous concussions **(E,F)**. The frequency at which PSD reached a peak amplitude (either 0.05 or 0.10 Hz) was used for sampling point estimates for *Coherence, Phase*, and *Gain*.

**Figure 3:**
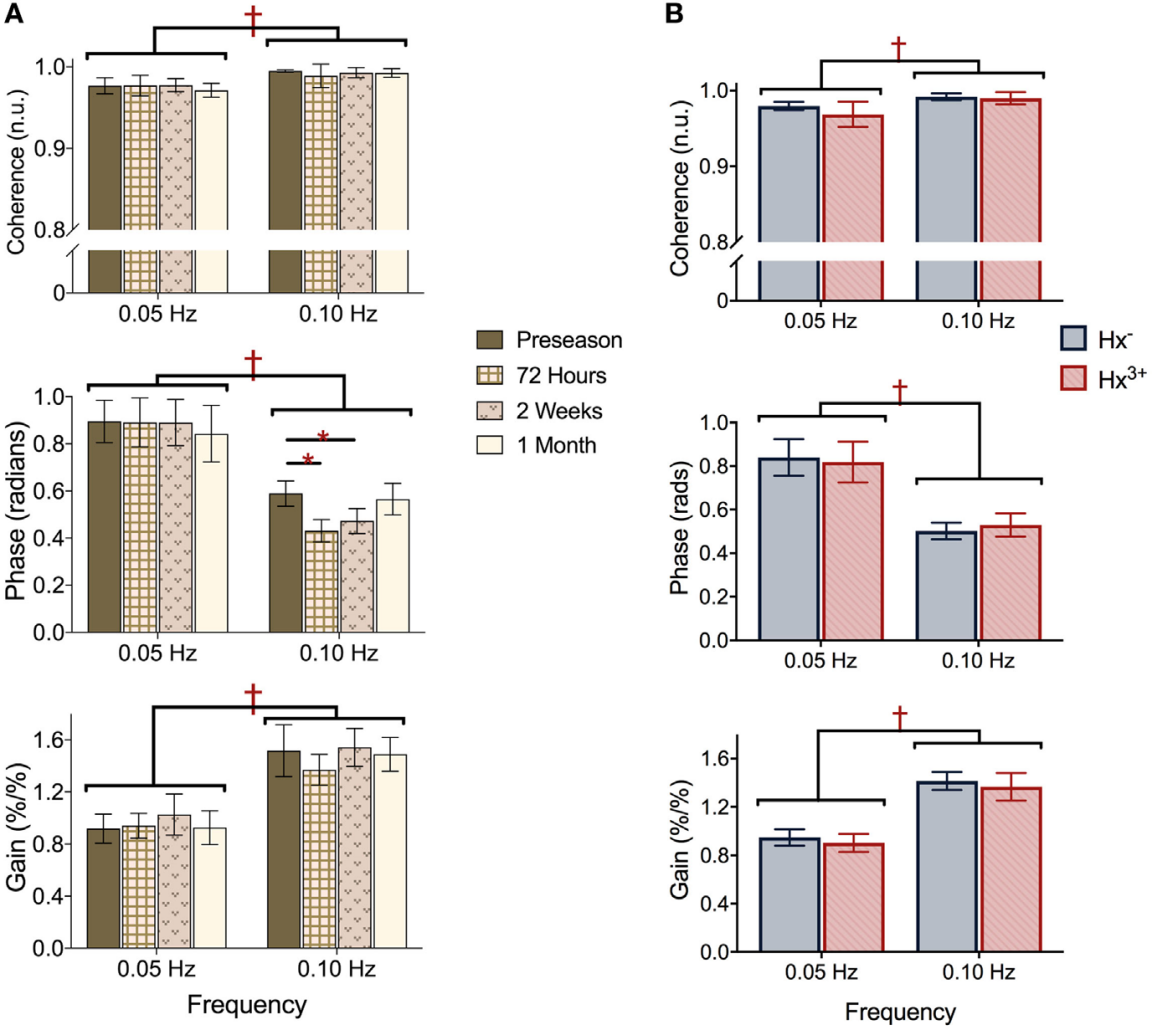
Transfer function analysis outcomes *Coherence* (top), *Phase* offset (middle), and normalized *Gain* (bottom) during squat–stand maneuvers assessed at **(A)** preseason and each post-concussion time point; **(B)** preseason in athletes with zero and three or more previous concussions. ^†^represents significant main effect of frequency (*p* all < 0.01), *denotes significant simple effect of time (*p* all < 0.01). Data presented as mean ± SE.

Representative time-series traces of BP, MCAv, and expired CO_2_ are presented in Figure 1 for the two different squat–stand frequencies. Figures 2A–D show the power spectrum density functions for MAP and MCAv in the acutely concussed athletes at different time points prior to and after their injury. Table 2 and Figure 3A displays the group averages for the transfer function analysis outcomes for *Coherence, Phase*, and *Gain*. A two-way RM-ANOVA indicated that both *Coherence* (*F*_1,17_ = 28.726, *p* < 0.001) and *Gain* (*F*_1,17_ = 102.329, *p* < 0.001) were significantly higher at 0.10 Hz than at 0.05 Hz, suggesting a preserved high-pass filter behavior of the cerebrovasculature following injury. Interaction terms between frequency and time were significant for *Phase* (*F*_3,51_ = 4.715, *p* = 0.007) and *Gain* (*F*_3,51_ = 2.866, *p* = 0.049), with subsequent analyses of simple effects revealing a significant effect of time for 0.10 Hz *Phase* (*F*_3,51_ = 8.061, *p* < 0.001). Relative to preseason, planned contrasts revealed significant *Phase* reductions (mean difference ± SEM) of 23% at 72 h (−0.136 ± 0.033 rads, *p* = 0.008) and 18% at 2 weeks (−0.105 ± 0.029 rads, *p* = 0.02) post injury, suggesting that the cerebrovascular response to changing BP was slower following concussion. By 1 month post injury, *Phase* offsets had recovered to values comparable to preseason (−0.039 ± 0.034 rads, *p* = 0.99). Furthermore, a significant correlation was found between *ΔPhase*_0.10 Hz_ from preseason to 72 h, and *ΔSAC* composite/total score from preseason to 72 h (*r* = 0.659, *p* = 0.026). However, correlations were not significant between *ΔPhase*_0.10 Hz_ and the change in symptom number (*r* = 0.277, *p* = 0.438), symptom severity (ρ = 0.212, *p* = 0.556), change in headache (ρ = 0.105, *p* = 0.745), change in “pressure in the head” (ρ = −0.171, *p* = 0.595), or change in performance on the BESS (*r* = 0.347, *p* = 0.268).

**Table 2:** Summary data for transfer function analysis outcomes in the concussed athletes across the four testing sessions.

Metric	Frequency (Hz)	Concussed athletes (*n* = 18)				*p*
		Preseason	72 h	2 Weeks	1 Month	
Coherence	0.05	0.978 (0.019)	0.979 (0.023)	0.977 (0.017)	0.972 (0.017)	0.923
	0.10	0.995 (0.003)	0.995 (0.005)	0.992 (0.014)	0.992 (0.012)	0.942

Phase (rads)	0.05	0.8911 (0.1962)	0.8984 (0.2300)	0.9030 (0.1724)	0.8622 (0.2485)	0.850
	0.10	0.5902 (0.1209)	0.4540 (0.0899)*	0.4853 (0.1129)*	0.5515 (0.1340)	**<*0.001***

Gain (%/%)	0.05	0.8913 (0.2012)	0.9137 (0.2017)	1.0536 (0.3354)	0.9027 (0.2815)	0.164
	0.10	1.4725 (0.4126)	1.3467 (0.2560)	1.5399 (0.2902)	1.4947 (0.2933)	0.227

*Coherence, correlation between BP and CBF; Phase, timing offset between BP and CBF; Gain, ratio of CBF amplitude to BP amplitude*.

*Data presented as mean (SD) unless otherwise noted*.

*Significant difference from preseason (Bonferroni-corrected t-test).

### Effect of Concussion History

Figures 2E,F show the power spectrum density functions for MAP and MCAv at baseline for participants with zero vs. three or more previous concussions. Table 3 and Figure 3B shows the transfer function analysis outcomes for the effects of concussion history on dCA. A two-way mixed ANOVA did not reveal main effects of concussion history for *Coherence* (*F*_1,52_ = 0.705, *p* = 0.405), *Phase* (*F*_1,52_ = 0.011, *p* = 0.917), or *Gain* (*F*_1,52_ = 0.174, *p* = 0.678). Significant main effects of frequency were observed for all variables (*p* always < 0.001), while interaction terms were nonsignificant (*p* = 0.191, 0.276, and 0.750 for *Coherence, Phase*, and *Gain*, respectively). As expected, *Coherence* (+1.74%, 95% CI = 0.01–0.024) and *Gain* (+51.6%, 95% CI = 0.408–0.549%/%) were higher, while *Phase* was lower (−39.6%, 95% CI = −0.271 to −0.400 rads) at 0.10 Hz relative to 0.05 Hz, indicating that lower-frequency BP oscillations were more effectively buffered than high-frequency oscillations (i.e., intact high-pass filter behavior).

**Table 3:** Summary preseason data for transfer function analysis outcomes in the athletes with 0 vs. 3+ previous concussions.

Metric	Frequency (Hz)	# Previous concussions		*p*
		0 (*n* = 42)	3+ (*n* = 31)	
Coherence	0.05	0.981 (0.016)	0.974 (0.027)	0.405
	0.10	0.993 (0.011)	0.995 (0.005)	

Phase (rads)	0.05	0.8653 (0.2574)	0.8246 (0.2305)	0.917
	0.10	0.4942 (0.1285)	0.5247 (0.1425)	

Gain (%/%)	0.05	0.9361 (0.2067)	0.9223 (0.2227)	0.678
	0.10	1.4261 (0.2196)	1.3896 (0.3392)	

*Coherence, correlation between BP and CBF; Phase, timing offset between BP and CBF; Gain, ratio of CBF amplitude to BP amplitude*.

*Data presented as mean (SD) unless otherwise noted*.

## Discussion

In the current report, we provide the first prospective study in the context of sport-related concussion to evaluate the capacity of the cerebral blood vessels to modulate blood flow in response to changes in BP. For this purpose, we used transcranial Doppler ultrasound and finger photoplethysmography to measure beat-to-beat CBF and systemic BP, respectively, during a squat–stand protocol. By this means, we were able to gain insight into the acute effects of sport-related concussion on dynamic cerebroautoregulation. In the discussion that follows, it is important to keep in mind the different tools used to measure the autoregulatory process and their limitations.

At a group level, key findings indicated transient post-injury impairments in the capacity of the cerebrovasculature to buffer BP oscillations occurring at 0.10 Hz, the timescale over which sympathetic contributions to CA are thought to operate (24, 28). At a within-subject level, more substantial impairments in CA were related with poorer performance on the SAC, a brief cognitive screen for concussion. Importantly, these impairments in pressure buffering persisted beyond medical clearance for return-to-full participation in contact sport in many cases, highlighting an important discrepancy between clinical (median 14 days) and physiological recovery (~30 days). However, when comparing preseason baseline data for contact sport athletes with a history of zero previous concussions to those with three or more previous concussions, no differences were observed in autoregulatory indices. These data support a process of transient concussion-induced physiologic dysfunction that does not appear to exert cumulative, lasting effects on the cerebrovasculature.

### CBF Alterations Post Concussion

Transient disruptions in CBF following concussion have been reported previously (6, 9, 10), although comparatively little study has been directed toward the mechanisms controlling CBF. In the earliest post-mTBI phase (<48 h), a period of acute hyperemia occurs with CBF peaking at 24 h (29), although this may be age-dependent (30, 31). Subsequently, evidence shows global and regional reductions in CBF, both subacutely (6, 9) and chronically (32). This has been demonstrated in both pediatric (9) and young adult (6) athlete populations. In both age groups, blood flow recovers to near-healthy athlete levels by ~30 days, although this may take longer in adolescents (9) and may be related to symptom resolution (6, 10). Despite this finding, when compared to athletes who had never sustained prior concussions, arterial spin labeling has shown that those with a history of concussion consistently display reduced frontotemporal CBF, independent of the number of previous concussions (33). In the current study, we did not observe significant reductions in resting MCAv as a function of concussion (Table 1). This may be due to the differences in sensitivity and spatial resolution between MRI and transcranial Doppler ultrasound to subtle changes in flow, with MRI providing a higher temporal resolution, whereas TCD provides an index of blood flow to all MCA-supplied territories. Despite our appreciation for the existence of CBF alterations following sport-related concussion, a paucity of evidence exists documenting their effects on the mechanisms governing the control of CBF. A comprehensive evaluation of cerebrovascular functioning should address three components, including reactivity to CO_2_, the neurovascular-coupling response, and an assessment of the cerebral pressure– flow relationship (34).

### Autoregulation Impairments in Traumatic Brain Injury

Autoregulatory mechanisms appear sensitive to acquired brain injury, with disruptions to the cerebral pressure–flow relationship being well documented following moderate and severe traumatic brain injury, with one small study suggesting similar deficits in a subset (8/29) of hospitalized mild TBI patients (17). Across multiple studies, 49–87% of severe TBI patients exhibit absent or impaired CA (35, 36). In alignment with our findings post concussion, the recovery of CA after severe TBI is delayed in some cases beyond 2 weeks (37). Disruptions in CA are predictive of outcome in acquired brain injury populations; in severe TBI, acutely compromised CA is a significant predictor or poor outcome (38); in patients with Fabry disease, impaired CA is thought to increase the risk of stroke (39); following subarachnoid hemorrhage, and CA dysfunction is among the primary factors predisposing patients to delayed cerebral ischemia and vasospasm at an individual level (40–42); impaired CA is an established independent risk factor for stroke (43). In the only other published study within the context of sport-related head trauma to consider CA, professional boxers have exhibited deficits in an index of CA compared to matched controls (44). The findings of the current study add to our understanding of the effects of acquired brain injury on CA, indicating that even comparatively mild brain injuries, such as concussions, may disrupt the ability of the brain to regulate CBF in the face of changing BP.

### Possible Mechanisms for CA Impairments Following Concussion

The current observation of reduced 0.10 Hz *Phase* offsets for at least 2 weeks post concussion implies an alteration in autonomic regulation of the cerebrovasculature that causes a delayed change in vascular resistance in response to a BP challenge. This complements previous reports of concussion-induced cardiovascular autonomic dysfunction and provides the first evidence that such autonomic dysregulation also affects cerebral hemodynamics. Concussion has been shown to alter cardiac autonomic function, particularly during exercise; altered heart rate variability patterns—associated with resting CBF (45)—suggest an increased sympathetic and/or a lowered parasympathetic tone for at least 10 days post injury (46–48). It has been suggested that the degree of autonomic uncoupling following acute brain injury is proportional to the severity of injury (49). The cerebrovascular tree is richly innervated by sympathetic fibers (50), with the large proximal cerebral arteries receiving dense innervation and primarily α-adrenergic receptors, while cerebral arterioles appear to be more richly populated with ß2-adrenergic receptors. Mounting evidence points to the importance of sympathetic innervation in the dynamic regulation of BP variability (51). Recently, pharmacological studies in healthy young adults have characterized sympathetic modulation of CA, whereby sympathetic activation has been shown to reduce both *Phase* and *Gain* at frequencies including 0.10 Hz, with even partial sympathetic blockade completely eliminating the *Phase* response (24, 28). In accordance with the suggestion that progressive impairment of autoregulation likely first affects the latency of the response (*Phase*) before affecting the efficiency (*Gain*) (52), we observed significant changes in *Phase* only. These results support a process of autonomic disruption affecting cerebral hemodynamics for at least 2 weeks post concussion, with resolution by 1 month, but did not reveal cumulative effects of multiple injuries.

The mechanism by which sport-related concussion may alter autonomic function, CA, and CBF remains unclear. Preclinical rodent models of TBI have shown reductions in both the number and diameter of capillaries at the injury site (53). Impaired CA has been associated with cerebral white matter damage (54). It is not definitively known whether vascular dysfunction precedes neuronal dysfunction or results from primary neuronal dysfunction (55). Whereas specifics underlying sympathetic modulation of cerebrovascular resistance remain poorly defined, brain injury models have demonstrated lateralized control of cardiac autonomic outflow from the cortex, in that left insular efferents exert parasympathetic effects and right insular efferents generate sympathetic responses (56–58). As such, disruption to the corpus callosum—the white matter structure most consistently reported to exhibit damage following concussion (7, 19)—could compromise balance between sympathetic and parasympathetic outflow (48). Furthermore, the Thayer model suggests that damage to prefrontal areas within the central autonomic network could exert a disinhibition of the central nucleus of the amygdala, resulting in a net increase in sympathetic activity through subsequent disinhibition of sympathoexcitatory neurons in the ventrolateral medulla (59). Such changes could cause a delay in vessel responsiveness to altered BP (i.e., slower change in resistance), which would generate the reduction in *Phase* observed in the current study, although this remains speculative. Whether the side and direction of the impact contributes to the pattern of cerebroautoregulatory responses observed is unclear. Modeling studies have certainly demonstrated strain and strain rate-dependent predictions of the location and extent of structural damage to neural tissue for a given impact force (60, 61). However, further study is required to demonstrate whether analogous localized damage takes place in the cerebrovasculature that may be linked to functional changes in CBF.

In contrast to the changes observed at the 0.10-Hz frequency, the dCA responses in the 0.05-Hz condition remained unaltered by acute concussion, implying that the myogenic contribution to cerebroautoregulation was not affected by the injury. This is in contrast to what has been observed previously in moderate to severe TBI (62) and suggests that myogenic tone is affected in an injury-severity-dependent manner.

### Limitations

There are several limitations to the current study. First, current clinical guidelines for concussion management dictate a period of complete physical and cognitive rest in the immediate post-injury phase. The potential detraining influence of strict rest on cerebrovascular control cannot be discounted; a single day of bed rest has been shown to reduce CBF for a substantial period of time (63). Recruiting a control group of healthy uninjured elite athletes to undergo a post-concussion rest and return-to-play protocol would allow insights into such an effect, but this is pragmatically unrealistic. Second, concussion remains a clinical diagnosis. As we were recruiting from multiple teams, there were three different physicians providing official concussion diagnoses. It is possible that the tendency toward diagnosis may have differed across providers, magnifying the omnipresent injury heterogeneity within our sample. Third, our sample consisted only of male athletes, primarily due to the availability of participants. This represents a significant shortcoming in the generalizability of our findings to the broader population, as females are reported to sustain higher rates of concussion and a greater time loss from concussion than their male counterparts (64). Fourth, the evaluation of acute effects of concussion benefited from a repeated-measures design to determine changes in CA. Insight into the cumulative effects of multiple concussions may be better gleaned using a similar longitudinal approach; substantial variability in “healthy” values of *Phase* and *Gain* may have precluded finding differences between Hx^−^ and Hx^3+^ groups due primarily to the between-subjects design. Lastly, TCD provides an index of CBF by measuring the velocity of red blood cells in the vessel of interest—that this relationship holds true requires an implicit assumption that the insonated vessel did not change diameter, which cannot be verified. However, there are ongoing debates surrounding the influences of MCA diameter changes on the evaluation of cerebral hemodynamics (65, 66). The effect of one of these modulators, P_CO2_, was assumed to be minimized as end tidals were tightly maintained during the protocol (Figure 1) (67). Furthermore, the utility of TCD to provide an estimate of CBF velocity independent of specific knowledge of the insonated vessel diameter has been demonstrated by a direct comparison to blood flow velocity responses quantified by perfusion MRI and arterial spin labeling (68). Despite these limitations, significant changes in dynamic CA seen in this study emphasize the need for more in-depth prospective investigations into the effects of sport-related concussion on mechanisms underlying the control of CBF.

## Conclusion

In order to mitigate both the incidence and the severity of concussive-type mild TBI, a better understanding of their neurobiological underpinnings is needed. This will facilitate the development of objective tools to improve the detection and management of these injuries. Although not currently appropriate for clinical application, TCD-based assessments of CA appear to be a promising approach to understanding the role of cerebral pressure buffering in the pathology underlying sport-related concussion. We detected significant reductions in the pressure-buffering capacity of the cerebral blood vessels of concussed athletes for at least 2 weeks following injury, which recovered by 1 month. While validation is required in a larger number of subjects, the results of this preliminary study support a process of transient autonomic disruption following concussion that may outlast symptom resolution and clinical recovery, encouraging the development of further prospective investigations into the effects of repetitive concussive and subclinical head impacts on mechanisms controlling CBF. Furthermore, the exploration of the relationships between age, sex, impact biomechanics, and CA integrity on susceptibility to injury, as well as between CA status and neurocognitive performance, is warranted.

## Ethics Statement

This study was carried out in accordance with the recommendations and approval of the University of British Columbia Clinical Research Ethics Board with written informed consent from all participants in accordance with the Declaration of Helsinki.

## Author Contributions

AW, JS, and PvD designed the study. AW, JS, and KB performed data collection, and AW, JS, SF, and MJ performed the analyses. All authors interpreted the data. AW wrote the manuscript. All authors had full access to the data and helped critically revise the manuscript before reviewing and approving the final version.

## Conflict of Interest Statement

The research described in this manuscript was conducted in the absence of any commercial or financial relationships that could be construed as a potential conflict of interest.
